# Antimicrobial susceptibility of *Neisseria gonorrhoeae* isolates from Hefei (2014–2015): genetic characteristics of antimicrobial resistance

**DOI:** 10.1186/s12879-017-2472-z

**Published:** 2017-05-25

**Authors:** Fa-Xing Jiang, Qian Lan, Wen-Jing Le, Xiao-Hong Su

**Affiliations:** 1 Department of Dermatology, Anhui Provincial Hospital, Hefei, 230001 China; 2 STD clinic, Institute of Dermatology, Chinese Academy of Medical Sciences & Peking Union Medical College, Nanjing, 210042 China

**Keywords:** *Neisseria gonorrhoeae*, Antimicrobial resistance, Azithromycin, Cephalosporins, Resistance determinants

## Abstract

**Background:**

Antimicrobial resistance (AMR) and genetic determinants of resistance of *N. gonorrhoeae* isolates from Hefei, China, were characterized adding a breadth of information to the molecular epidemiology of gonococcal resistance in China.

**Methods:**

126 *N. gonorrhoeae* isolates from a hospital clinic in Hefei, were collected between January, 2014, and November, 2015. The minimum inhibitory concentration (MIC) of *N. gonorrhoeae* isolates for seven antimicrobials were determined by the agar dilution method. Isolates were tested for mutations in *penA* and *mtrR* genes and 23S rRNA, and also genotyped using *N. gonorrhoeae* multi-antigen sequence typing (NG-MAST).

**Results:**

All *N. gonorrhoeae* isolates were resistant to ciprofloxacin; 81.7% (103/126) to tetracycline and 73.8% (93/126) to penicillin. 39.7% (50/126) of isolates were penicillinase producing *N. gonorrhoeae* (PPNG), 31.7% (40/126) were tetracycline resistant *N. gonorrhoeae* (TRNG) and 28.6% (36/126) were resistant to azithromycin. While not fully resistant to extended spectrum cephalosporins (ESCs), a total of 14 isolates (11.1%) displayed decreased susceptibility to ceftriaxone (MIC ≥ 0.125 mg/L, *n* = 10), cefixime (MIC ≥ 0. 25 mg/L, *n* = 1) or to both ESCs (*n* = 3). *penA* mosaic alleles XXXV were found in all isolates that harbored decreased susceptibility to cefixime, except for one. Four mutations were found in *mtrR* genes and mutations A2143G and C2599T were identified in 23S rRNA. No isolates were resistant to spectinomycin. Gonococcal isolates were distributed into diverse NG-MAST sequence types (STs); 86 separate STs were identified.

**Conclusions:**

*N. gonorrhoeae* isolates from Hefei during 2014–2015, displayed high levels of resistance to antimicrobials that had been recommended previously for treatment of gonorrhea, e.g., penicillin, tetracycline and ciprofloxacin. The prevalence of resistance to azithromycin was also high (28.6%). No isolates were found to be fully resistant to spectinomycin, ceftriaxone or cefixime; however, 11.1% isolates, overall, had decreased susceptibility to ESCs.

## Background

*Neisseria gonorrhoeae* is the etiologic agent of gonorrhea, one of the most common bacterial sexually transmitted infections (STIs) worldwide. WHO estimated that 78.3 million of new cases of gonorrhea occurred among adults globally in 2012 [1].100, 245 cases of gonorrhea were reported nationally by the China Centers for Disease Control and Prevention in 2015, making it the fifth most commonly reported communicable disease in China [2]. At this time there are no effective vaccines for gonococcal infections and antimicrobial treatment continues to be the mainstay of control. However, *N. gonorrhoeae* has developed resistance to antimicrobials that had been used previously for treatment of this infection including sulfonamides, penicillins, tetracyclines and quinolones. Presently, extended spectrum cephalosporins (ESCs) that include ceftriaxone and cefixime are recommended as the first-line treatment of gonorrhea in most parts of the world. In many countries azithromycin has been added not just to treat chlamydia infection that often co-infects, but also to supplement treatment for *N. gonorrhoeae* itself [3]. Diminished susceptibility of *N. gonorrhoeae* and emergence of full resistance and treatment failures with ESCs (mainly pharyngeal gonorrhea) have been documented in several countries [4]. Furthermore, *N. gonorrheae* strains with high-level azithromycin resistance have been reported in France [5], the United States [6], Australia [7] and China [8]. Resistance to azithromycin threatens efficacy of dual antimicrobial therapy (ESCs plus azithrothmycin) that may result in decreased treatment options and enhance the possibility of untreatable infection.

A key component of a successful response plan to gonococcal antimicrobial resistance (AMR) is to conduct timely surveillance of resistance and treatment failures across geographic regions (e.g. GISP [U.S] and GASP (WHO/Europe) [9, 10]) and to characterize the genetic elements of resistant strains. Antimicrobial susceptibility patterns vary by geographic region. AMR surveillance programs have been conducted in several cities in China [11, 12]. The aims of the present study were to describe the prevalence of gonococcal AMR and the molecular epidemiological characteristics of *N. gonorrhoeae* strains from 2014 to 2015 in Hefei, a city in eastern China.

## Methods

### Clinical isolates

Clinical isolates of *N. gonorrhoeae* (*n* = 126) investigated in this study were collected consecutively from men with urethritis (urethral discharge and/or dysuria) and women with cervical infection in the STD clinic at Anhui Provincial Hospital, China, between January, 2014 and November 2015. Cotton swabs, used to obtain cervical and urethral specimens, were immediately streaked onto Thayer-Martin (T-M) selective medium to isolate *N. gonorrhoeae*. Inoculated plates were incubated at 36 °C in 5% carbon dioxide for 24–48 h. and *N. gonorrhoeae* was identified by colonial morphology, Gram’s stain, and oxidase testing. Gonococcal strains were subcultured onto chocolate agar plates, preserved in tryptone-based soy broth and then stored at −70 °C until used.

### Antimicrobial susceptibility testing

Minimum inhibitory concentrations (MICs; mg/L) of *N. gonorrhoeae* isolates to penicillin, tetracycline, ciprofloxacin, spectinomycin, azithromycin, ceftriaxone and cefixime were determined on Difco GC medium base agar supplemented with 1% BBL™, IsoVitaleX™ enrichment (Becton, Dickinson and Company) using the agar dilution method recommended by the Clinical and Laboratory Standards Institute (CLSI) [13]. Gonococcal isolates were subcultured from frozen stocks onto chocolate agar and the resulting colonies were re-subcultured at 36 °C in 5% CO_2_ for 18–20 h before antimicrobial susceptibility testing was performed. Concentrations of antibiotics used were: penicillin, 0.06–8 mg/L; tetracycline, 0.125–16 mg/L; ciprofloxacin, 0.06–8 mg/L; spectinomycin, 4–128 mg/L; azithromycin,0.015–2048 mg/L; ceftriaxone, 0.002–0.5 mg/L and cefixime,0.002–0.5 mg/L. All antibiotics were purchased from Sigma Aldrich (USA), except for azithromycin, which was purchased from Shanghai yuanye Bio-Technology Co., Ltd. 95% ethanol was used to dissolve azithromycin powder to obtain a stock solution of 4096 mg/L; azithromycin stock was further diluted with distilled water to prepare twofold working dilutions. ATCC49226 and WHO reference strains G, K, M, O, P were used as quality controls for MIC determinations. Results were interpreted according to the CLSI standard, except for azithromycin that used breakpoints recommended by the European Committee on Antimicrobial Susceptibility Testing (EUCAST); www.eucast.org [14].Criteria for decreased susceptibility to ceftriaxone (MIC ≥ 0.125 mg/L) and cefixime (MIC ≥ 0.25 mg/L) were defined by WHO [9].

### Identification and typing of β-lactamase and *tetM* encoding plasmids

β-lactamase production was determined by the paper acidometric method [15]. Isolates were classified as high level resistant to tetracycline (TRNG) if MICs were ≥16 mg/L and resistant (plasmid-mediated) to penicillin (PPNG) if they were β-lactamase positive [16]. The type of β-lactamase and *tetM* encoding plasmids were determined by PCR [17].

### Genetic determinants associated with decreased susceptibility to ESCs and resistance of *N. gonorrhoeae*to azithromycin

Amplification of *penA* and *mtrR* genes and 23S rRNA were performed using published primers and conditions [18–20]. 14 gonococcal isolates with decreased susceptibility to ceftriaxone or cefixime were tested for *penA* mutations, including 3 isolates that had decreased susceptibility to both cefixime and ceftriaxone; all isolates were tested for mutations in *mtrR* and 41strains (including all 36 azithromycin-resistant and 5 randomly selected azithromycin sensitive strains) were tested for 23S rRNA mutations. PCR products were sequenced twice in both directions using an Applied Biosystems 3730XL DNA automatic sequencer. The nucleotide and deduced amino acid sequences were analyzed using the EditSeq program (LaserGene software [version 7.1; DNAStar Corp.]) and aligned against their respective prototypes using the Megalign program (LaserGene software). Standard sequences used for alignment in the study were: PBP2; wild strain LM306 (GenBank accession no. M32091 [18]); 4 alleles of 23S rRNA (GenBank accession no. AF450074 to AF450081 [20]) and mtrR (GenBank accession no. Z25796 [19]).

### Molecular epidemiologic typing

*N. gonorrhoeae* multiantigen sequence typing (NG-MAST) was performed on all isolates (*n* = 126) as described [21]. The allele numbers of *por* and *tbpB*, and the sequence types (STs) were assigned using the NG-MAST website (www.ng-mast.net).

### Statistical analysis

Statistical analysis was performed using statistical software IBM SPSS Statistics version 19.0 for comparisons of proportions. The level of significance was set at *P* < 0.05.

## Results

### Antimicrobial susceptibility testing

Antimicrobial susceptibility testing of 126 gonococcal isolates obtained from Hefei, China, between January, 2014 and November, 2015, is indicated in Table 1. Resistance to penicillin and tetracycline was 73.8% (93/126) and 81.7% (103/126), respectively; all isolates were resistant to ciprofloxacin. 28.6% (36/126) of isolates were resistant to azithromycin; among them,36.1% (13/36) displayed high-level azithromycin resistance (MIC ≥ 256 mg/L). No isolates were found to be fully resistant to spectinomycin, ceftriaxone or cefixime. However,14 isolates (11.1%) displayed decreased susceptibility to ceftriaxone (MIC ≥ 0.125 mg/L, *n* = 10), cefixime (MIC ≥ 0. 25 mg/L, *n* = 1) or to both ESCs (*n* = 3).Two azithromycin-resistant isolates also exhibited reduced susceptibility to ceftriaxone. Overall, plasmid mediated resistance to either penicillin or tetracycline was exhibited by 60.3% (76/126) of isolates: 39.7% (50/126) were PPNG; 31.7% (40/126) were TRNG and 11.1% (14/126) were PPNG/TRNG. 80% (40/50) PPNG isolates carried the Asia type β-lactamase encoding plasmid and 20% (10/50) harbored the African type plasmid. No Toronto plasmid was detected. 97.5% (39/40) of TRNG isolates carried the Dutch type *tetM* containing plasmid and the remainder carried the American-type.

**Table 1:** Antimicrobial susceptibility of *Neisseria gonorrhoeae* isolates (*n* = 126) from Hefei, China (2014–2015)

Antimicrobial breakpoints (susceptible/resistant [mg/L])	Number			MIC (mg/L)		
	Susceptible	Intermediate	Resistant	Range	MIC_50_	MIC_90_
Penicillin G (S ≤ 0.06/*R* ≥ 2)	0	33	93	0.125 to >8	2	>8
Tetracycline (S ≤ 0.25/*R* ≥ 2)	2	21	103	0.25 to >16	4	>16
Ciprofloxacin (S ≤ 0.06/*R* ≥ 1)	0	0	126	0.25 to >8	>8	>8
Azithromycin(S ≤ 0.25/*R* ≥ 1)	48	42	36	≤0.015 to >2048	0.5	> 2048
Spectinomycin (S ≤ 32/*R* ≥ 128)	126	0	0	8 to 32	16	32
Cefixime (S ≤ 0.25)	126	0	0	0.004 to 0.25	0.03	0.06
Ceftriaxone(S ≤ 0.25)	126	0	0	0.004 to 0.25	0.06	0.125

*MIC* minimum inhibitory concentration

### Characterization of resistance in *penA* and *mtrR* genes and 23S rRNA

#### Mutations in the *penA* gene

We sequenced the *penA* gene from 14 isolates that displayed decreased susceptibility to ceftriaxone (*n* = 10), cefixime (*n* = 1) or to both ESCs (*n* = 3) (Table 2). Six PBP2 amino acid sequence patterns were identified, including a mosaic allele (XXXV) [18]. PBP2 allele XIII [19] was the predominant type (*n* = 5), followed by mosaic allele XXXV (*n* = 3) and alleles XVIII (*n* = 3), V (*n* = 1), XVII (*n* = 1) and XXI (*n* = 1) [19, 22, 23]. The three isolates that harbored mosaic allele XXXV had decreased susceptibility to cefixime; one (of the 3) displayed decreased susceptibility to both ceftriaxone and cefixime. Among 11 non-mosaic isolates that included 10 with decreased susceptibility to ceftriaxone and 1 to both ESCs, seven harbored alleles XIII, XVII and XXI and displayed an A501V mutation in PBP2. Three isolates with allele XVIII possessed an A501T mutation and one had a G542S mutation.

**Table 2:** Characteristics of *N gonorrhoeae* isolates (*n* = 14) with increased MICs to ceftriaxone (MIC, 0.125 mg/L) or cefixime (MIC, 0.25 mg/L)

Strain number	MIC(mg/L)		PBP2 allele	NG-MAST
	Ceftriaxone	Cefixime		
HF17	0.125	0.06	XVIII	ST12197^b^
HF20	0.125	0.25	XIII	ST1577
HF37	0.125	0.25	XXXV^a^	ST12200^b^
HF 42	0.125	0.125	XIII	ST12192^b^
HF 109	0.06	0.25	XXXV^a^	ST12647^b^
HF 141	0.125	0.125	XIII	ST12662^b^
HF 149	0.125	0.125	XIII	ST12192^b^
HF 152	0.125	0.06	V	ST12198^b^
HF 154	0.125	0.06	XIII	ST 2318
HF 156	0.125	0.25	XXXV^a^	ST13044^b^
HF 163	0.125	0.06	XVIII	ST3356
HF 169	0.125	0.06	XVII	ST13133^b^
HF 170	0.125	0.125	XXI	ST13134^b^
HF 175	0.125	0.06	XVIII	ST12479^b^

^a^Mosaic penA alleles

^b^Novel STs

#### Mutations in the *mtrR* gene

All 126 isolates had mutations in the *mtrR* gene. Adenine (A) was deleted in the promoter region of *mtrR* in 107(87.3%) strains; there was no difference (*p* = 0.181) in isolates with MICs ≥1 mg/L to azithromycin (33/36 [91.7%]) compared to isolates with an MIC ≤0.5 mg/L (74/90 [82.2%]). However, the G45D mutation in the *mtrR* gene was identified more often in azithromycin-resistant isolates (25/36 [69.4%]) than in susceptible isolates (9/90 [10%]) (*p* < 0.01). The A40D mutation was present in three isolates with (lower) azithromycin MICs of 0.125 mg/L; these isolates also exhibited reduced cefixime and/or ceftriaxone susceptibility. The A39T mutation was found in 16 more sensitive isolates, having MICs to azithromycin >0.125 mg/L but <0.5 mg/L.

#### Mutations in domains of 23S rRNA

All 36 azithromycin-resistant isolates and five –susceptible isolates were tested for mutations in 23S rRNA. The A2143G (*N. gonorrhoeae* numbering) mutation was identified in four alleles in the13 isolates with high-level azithromycin resistance (MICs ≥ 256 mg/L). AC259 9 T mutation in four alleles was found in four isolates (MICs, 8 to 32 mg/L).No mutations were found in the five azithromycin-susceptible isolates.

### Sequence-based molecular epidemiologic typing

The 126 *N. gonorrhoeae* isolates were assigned to 86 different NG-MASTs (abbreviated henceforth as STs); 53 (61.6%) STs were novel. 19 STs were shared by ≥ 2 isolates and 67 STs were represented only in single isolate. The most common ST was7469, represented by 6 isolates (4.8%), followed by ST1866 (5 isolates; 4.0%). All ST7469 isolates were comprised exclusively of the Dutch-type TRNG; all ST1866 isolates were resistant to azithromycin (MIC ≥ 1 mg/L).Three isolates, which possessed the *penA* mosaic allele XXXV and had decreased susceptibility to cefixime (MIC = 0.25 mg/L), belonged to different STs (ST12200, ST12647, ST13044) but shared identical *tbpB*10.

## Discussion

A high prevalence of resistance to antimicrobials previously used for the treatment of gonorrhea was documented in Hefei (2014–15) in this study: penicillin (68.9%); tetracycline (80.6%) and ciprofloxacin (100%). Similar levels of resistance to ciprofloxacin, tetracycline and penicillin have been reported in *N. gonorrhoeae* isolates from other cities in China: Nanjing (penicillin 67.7%, tetracycline 97.9% and ciprofloxacin 98.8%, in 2011–2012) [17]; Shanghai (penicillin 90%, tetracycline 82.56% and ciprofloxacin 100%, in 1988–2013) [11] and Guangzhou (penicillin 90.1% and ciprofloxacin 98%, in 2008–2013) [12]. A high percentage of PPNG and TRNG isolates was also identified in Hefei. Because penicillin and tetracycline are no longer used for the treatment of gonorrhea in China, continued high resistance may have been the result of having used these antimicrobials to treat other infections, particularly in patients who self-medicate, which is common in China [24].

Resistance to azithromycin was 28.6%, higher than had been reported from certain other countries, such as Japan [25] and Spain [26] during this period. We found that 10.3% (13/126) of isolates were highly resistant to azithromycin (MIC > 2048 mg/L). *N. gonorrhoeae* isolates with high-level azithromycin resistance have also been reported from other Chinese cities; Hanghzou [27] and Guangzhou [28]. Similar to other reports [29, 30], our isolates with high-level azithromycin resistance all contained the mutation A2143G in 4 alleles in the peptidyltransferase loop of domain V of 23S rRNA. The C2599T mutation was found in 23S rRNA alleles in four moderately azithromycin-resistant *N. gonorrhoeae* isolates (representing MICs of 8, 16, and 32 mg/L).

There are no reports of ceftriaxone treatment failures in China. The percentage of isolates with decreased susceptibility to ceftriaxone (MIC ≥ 0.125 mg/L) was 10.3% (13/126) in Hefei in 2014–2015, which is higher than the corresponding 4.5% reported from another eastern Chinese city, Nanjing, between 2011 and 2012 [17]. The proportions of ceftriaxone non-susceptible isolates (MIC ≥ 0.125 mg/L) in Shanghai, the largest city in eastern China, were 7%–13% from 2011 to 2013 [11]. This suggests the possibility that resistance to extended spectrum cephalosporins (ESCs) may be increasing in eastern China. PBP2 is targeted by cephalosporins and mutations in PBP2 may lead to a decline in susceptibility to ESCs [31]. Four (3.2%) of our isolates had reduced susceptibility to cefixime (MIC = 0.25 mg/L); three (ST12200, ST12647, ST13044) had the mosaic allele XXXV. The majority (7/11 [63.6%]) of non-mosaic isolates with decreased susceptibility to ceftriaxone harbored mutations in PBP2 at position 501 (A501V or A501T). Other studies have also found that A501 mutations in non-mosaic *penA* alleles play an important role in decreased sensitivity to ESCs [32]. A G542S mutation in PBP2 has been tentatively linked epidemiologically to higher ceftriaxone MICs [33] but an absolute correlation with a *penA* fixed-point mutation has not been shown. ST1407 *N. gonorrhoeae* strains, which display decreased susceptibility to ESCs or full resistance [34], and have spread worldwide, were not identified in Hefei. Five different STs (ST10367, ST12649, ST12650, ST12657, ST13132) that shared the same *tbpB*110 with ST1407 were identified but their *por* alleles were different (by 13 to 37 nucleotides) than *por*908 of ST1407; all isolates with these STs were susceptible to both ceftriaxone and cefixime. ST1407, however, has been isolated in Shanghai [35].

Significant diversity of isolates was identified among 126 isolates that were divided into 86 NG-MAST STs. Notably, ST1866 has been reported only from China and identified to be associated with high level azithromycin resistance [28]. The 5 ST1866 isolates in this study were azithromycin-resistant strains; three had high level resistance to azithromycin.

## Conclusions

This is the first study that describes antimicrobial resistance profiles and molecular determinants of *N. gonorrhoeae* strains isolated from Hefei, China. *N. gonorrhoeae* isolates from Hefei, during 2014–2015, possessed high level resistance to antimicrobials previously recommended for the treatment of gonorrhea, e.g., penicillin, tetracycline and ciprofloxacin. Azithromycin-resistance was documented in 28.6% of isolates, higher than that reported from other areas of the world. No isolates were found to be resistant to ceftriaxone, cefixime or spectinomycin. However, 11.1% isolates had decreased susceptibility to ESCs, which was often associated with mutations in the *penA* gene. Gonococcal isolates were distributed into diverse NG-MAST sequence types.

## Abbreviations

AMR: Antimicrobial resistance
CLSI: Clinical and Laboratory Standards Institute
ESCs: Extended-spectrum cephalosporins
GASP: Gonococcal antimicrobial surveillance programme
GISP: Gonococcal Isolate Surveillance Project
MIC: Minimum inhibitory concentration
NG-MAST: *Neisseria gonorrhoeae* multi-antigen sequence typing
PBP: Penicillin-binding protein
PPNG: Penicillinase-producing *N. gonorrhoeae*
ST: Sequence type
STD: Sexually transmitted disease
STI: Sexually transmitted infection
TRNG: Tetracycline-resistant *N. gonorrhoeae*
WHO: World Health Organization
